# Association of neonatal outcome with birth weight for gestational age in Chinese very preterm infants: a retrospective cohort study

**DOI:** 10.1186/s13052-024-01747-1

**Published:** 2024-10-04

**Authors:** Liangliang Li, Jing Guo, Yanchen Wang, Yuan Yuan, Xing Feng, Xinyue Gu, Siyuan Jiang, Chao Chen, Yun Cao, Jianhua Sun, Shoo K Lee, Wenqing Kang, Hong Jiang, The Chinese Neonatal Network, Xing Feng, Chao Chen, Yun Cao, Jianhua Sun, Wenqing Kang, Hong Jiang, Shoo K. Lee, Lizhong Du Du, Wenhao Zhou, Falin Xu, Xiuying Tian, Huayan Zhang, Yong Ji, Zhankui Li, Bin Yi, Xindong Xue, Chuanzhong Yang, Dongmei Chen, Sannan Wang, Ling Liu, Xirong Gao, Hui Wu, Changyi Yang, Shuping Han, Ruobing Shan, Gang Qiu, Xinnian Pan, Youyan Zhao, Mingxia Li, Xuqiang Ye, Lili Wang, Jiangqin Liu, Zhenlang Lin, Yuan Shi, Xiuyong Cheng, Jiahua Pan, Qin Zhang, Qin Zhou, Long Li, Pingyang Chen, Xiaoying Li, Ling Yang, Deyi Zhuang, Yongjun Zhang, Jinxing Feng, Li Li, Xinzhu Lin, Yinping Qiu, Kun Liang, Li Ma, Liping Chen, Liyan Zhang, Hongxia Song, Zhaoqing Yin, Mingyan Hei, Huiwen Huang, Jie Yang, Dong Li, Guofang Ding, Jimei Wang, Qianshen Zhang, Xiaolu Ma, Joseph Ting

**Affiliations:** 1 Division of Neonatology, The Affiliated Hospital of Qingdao University, NO. 16 Jiang Su Street, Qingdao, Shandong Province 266000 China; 2 Division of Neonatology, Children’s Hospital Affiliated of Zhengzhou University, Henan Children’s Hospital, No.33 Longhu East Road, Zhengzhou, Henan 450018 China; 3 Departments of Obstetrics & Gynaecology, and Health Research Methods, Evidence, and Impact, McMaster University, Hamilton, Canada; 4 Division of Neonatology and Center for Newborn Care, Guangzhou Women and Children’s Medical Center, Guangdong, China; 5 Division of Neonatology, Children’ Hospital of Soochow University, Jiangsu, China; 6 National Health Commission Key Laboratory of Neonatal Diseases, Fudan Unviersity, Children’s Hospital of Fudan University, Shanghai, China; 7 Division of Neonatology, Children’s Hospital of Fudan University, Shanghai, China; 8 Division of Neonatology, Shanghai Children’s Medical Center, Shanghai Jiao Tong University School of Medicine, Shanghai, China; 9 Maternal-Infant Care Research Center and Department of Pediatrics, Mount Sinai Hospital, Toronto, Canada

**Keywords:** Birth weight for gestational age, NICU, SGA, Very preterm infants, Health outcomes

## Abstract

**Background:**

The neonatal outcomes across different percentiles of birth weight for gestational age are still unclear.

**Methods:**

This retrospective cohort study was conducted within 57 tertiary hospitals participating in the Chinese Neonatal Network (CHNN) from 25 provinces throughout China. Infants with gestational age (GA) 24^+0^-31^+6^ weeks who were admitted within 7 days after birth were included. The composite outcome was defined as mortality or any one of neonatal major morbidities, including necrotizing enterocolitis (NEC), bronchopulmonary dysplasia (BPD), severe intraventricular hemorrhage (IVH), cystic periventricular leukomalacia (cPVL), severe retinopathy of prematurity (ROP), and sepsis. Multivariable logistic regressions using generalized estimating equation approach were conducted.

**Results:**

A total of 8380 infants were included with a mean GA of 30 (28–31) weeks. Of these, 1373 (16.5%) were born at less than 28 weeks, while 6997 (83.5%) had a GA between 28 and 32 weeks. Our analysis indicated that the risk of composite outcomes was negatively associated with birth weight for gestational age, and compared to the reference group, the multiple-adjusted ORs (95%CI) of composite outcomes were 4.89 (3.51–6.81) and 2.16 (1.77–2.63) for infants with birth weight for gestational less than 10th percentile and 10th -30th percentile, respectively. The ORs (95%CI) of mortality, NEC, BPD, severe ROP, and sepsis in infants with birth weight for gestational age at 10th-30th percentile were 1.94 (1.56–2.41), 1.08 (0.79–1.47), 2.48 (2.03–3.04), 2.35 (1.63–3.39), and 1.39 (1.10–1.77), respectively.

**Conclusion:**

Our study suggested that the risk of adverse neonatal outcomes increased significantly when the birth weight for gestational age was below the 30th percentile. Regular monitoring and early intervention are crucial for these high-risk infants.

**Supplementary Information:**

The online version contains supplementary material available at 10.1186/s13052-024-01747-1.

## What is already known on this topic

SGA as risk factor for morbidity and mortality of preterm infants has been well confirmed by previous research.

## What this study adds

The risk of adverse neonatal outcomes increased significantly when the birth weight for gestational age was below the 30th percentile.

## How this study might affect research, practice or policy

Regular monitoring and early intervention are crucial for these high-risk infants. By enhancing follow-up care, we can promptly identify and manage potential health issues, thereby improving the long-term health outcomes of these infants.

## Background

Growth restricted very preterm newborns have a higher risk of mortality, morbidity, and postnatal growth retardation [1], with adverse effects on their cognitive or physical development [2]. Clinically, weight for gestational age is a common indicator of fetal development, which is usually classified into large- or small-for-gestational-age birthweight (LGA & SGA) based on growth standards using 10th and 90th population-based centiles [3, 4]. Although SGA as a risk factor for morbidity and mortality of preterm infants has been well confirmed by previous research [5–7], the ambiguities of its accuracy in the cut-off points persist. Recently, Choi et al. indicated that the diagnostic accuracy of SGA defined by birthweight centiles varied considerably for detecting different adverse neonatal outcomes in both term and preterm deliveries. For the Fenton-birth weight standard, using < 25th centile to define preterm-SGA might be the best for detecting composite morbidity (AUC: 0.566), while for perinatal death, < 10th centile might be the best performing threshold (AUC: 0.634) [8]. A previous large-scale study stratified infants into smaller subsets than conventional definition (SGA, LGA, and AGA), and suggested that infants with a birthweight below the 50th percentile are at higher risk of perinatal mortality than those with an array of 50th -90th percentile [9]. Hendrix et al. have also shown that some of the newborns considered suitable for gestational age also exhibit suboptimal fetal development and are associated with adverse neonatal outcomes [10]. Considering using the conventional 10th centile as the cutoff point might miss infants at risk, J. Zeitlin et al. explored the association between detailed birthweight centiles and risk of morbidities or mortality. They have pointed out that infants at less than the 25th centile of birth weight have a higher risk of mortality and bronchopulmonary dysplasia [11]. Although several potential cutoff points have been proposed in previous studies, some challenges still underline when applying those certain thresholds to different ethnicities and countries. Moreover, providing appropriate resources and intervention strategies for perinatal emergencies and preterm infants can reduce mortality and adverse outcomes [12, 13]. Therefore, it is necessary to explore the relationship between the detailed percentile of birth weight for gestational age and adverse neonatal outcomes.

In this study, we aimed to investigate the baseline data related to detailed birth weight percentiles for gestational age among very preterm infants, to discover their association with neonatal outcomes in China and to provide appropriate management for the very preterm infants. Furthermore, considering some causes of preterm birth, such as maternal hypertension, are more likely to be related to growth restriction [14, 15], we also explored the possible interaction effect between maternal hypertension and growth restriction.

## Methods

### Data source

We conducted a retrospective cohort study using the Chinese Neonatal Network (CHNN) databases. The CHNN has established and maintained a standardized clinical database of preterm infants < 32 weeks’ gestation or < 1500 g in participating neonatal intensive care units (NICUs) throughout China, aiming to monitor outcomes and care practices. A total of 57 hospitals from 25 provinces throughout China collected whole-year data using the CHNN database in 2019. These 57 hospitals included four national children’s medical centers, 4 regional children’s medical centers, and 30 provincial perinatal or children’s medical centers. The other 19 hospitals comprised major referral centers in large cities across China. Among them, 43 hospitals were perinatal centers with birthing facilities, and 14 hospitals were free-standing children’s hospitals. All hospitals could provide complicated care for infants < 32 weeks gestation. Detailed information about the CHNN can be found elsewhere [16].

This study was approved by the ethics review board of Children’s Hospital of Fudan University (2018 − 296), which was recognized by all participating hospitals. Waiver of consent was granted at all sites since only de-identified patient data were accessed. All methods were performed in accordance with the ethical standards as laid down in the Declaration of Helsinki.

### Study population

Infants with GA from 24^+0^ to 31^+6^ weeks admitted to CHNN sites within 7 days after birth were included in this study and were followed up until discharge. We excluded infants with stillbirth, delivery room death, unknown sex, or congenital malformations. We also excluded infants who were transferred to non-participating hospitals within 24 h after birth.

### Data collection

Data were collected by well-trained data collectors and then entered into a secure online database with in-built error checking and a standard manual of operations. The patients’ identity information was kept confidential. Plausibility checks of incoming data are performed routinely to ensure the quality of data.

### Exposure

Based on the Fenton curve, birth weight percentiles for gestational age is the percentile of birth weight corresponding to each gestational age, and the Z-score of birth weight was calculated in each infant using the Lambda, Mu, and Sigma statistics methods. Birth weight for gestational age was then computed by area of standard normal distribution [17]. The birth weight for gestational age was then classified into 6 categories, including birth weight less than the 10th percentile (SGA), 10th -30th percentile, 30th -50th percentile, 50th -70th percentile, 70th -90th percentile, and more than 90th percentile (LGA).

### Outcomes

The composite outcome was defined as mortality or any one of the major morbidities, including necrotizing enterocolitis (NEC), bronchopulmonary dysplasia (BPD), severe intraventricular hemorrhage (IVH), cystic periventricular leukomalacia (cPVL), severe retinopathy of prematurity (ROP), and sepsis. Severe IVH was defined as ≥ grade 3 according to the Papile’s criteria [18]. Cystic PVL was defined as the presence of periventricular cysts on cranial ultrasound or MRI [19]. NEC was defined according to Bell’s criteria [20, 21]. Sepsis was defined as positive blood or cerebrospinal fluid culture and antibiotic therapy or intent of antibiotics therapy ≥ 5 days. Early-onset sepsis was defined as sepsis that occurred within 72 h after birth [22]. Late-onset sepsis was defined as sepsis that occurred after 72 h after birth. ROP was diagnosed according to the International Classification of ROP [23]. BPD was defined as ventilation or oxygen dependency at 36 weeks’ postmenstrual age or discharge if before 36 weeks [24].

### Covariates

Gestational age was determined using the hierarchy of best obstetric estimates based on prenatal ultrasound, menstrual history, and obstetric examination. If the obstetric estimate was not available or was different from the postnatal estimate of gestation by more than two weeks, the gestational age was estimated using the Ballard score [25]. Usage of antenatal corticosteroids was defined as maternal receipt of at least one dose of dexamethasone or betamethasone before delivery. Prenatal care was defined as ≥ 1 pregnancy-related hospital visits during pregnancy. The transport risk index of physiologic stability (TRIPS) score was performed as an illness severity score on NICU admission [26].

### Statistical analysis

Chi-square test or ANOVA was applied to determine the difference in infant and maternal characteristics across 6 categories of birth weight for gestational age.

We compared the incidence of neonatal outcomes between the 6 groups of birth weight for gestational age using the Chi-square test. The Cochran-Armitage test for trend was conducted to assess the trend of incidence of neonatal outcomes among the exposures group. To further assess the influence of different birth-weight-for gestational age on neonatal outcomes, we compared the neonatal outcomes using multivariate logistic regressions for correlated data, adjusted for potential confounders identified from baseline descriptive analysis. Potential confounders included maternal age, antenatal steroid usage, maternal hypertension, maternal diabetes, cesarean-section, primiparity, multiple birth, gestational age, infant’s gender, and birthplace. Model parameters were estimated with a generalized estimating equation (GEE) approach with a compound symmetric covariance structure to account for clustering effects within hospitals. Stratified analysis was conducted within different GA groups and maternal hypertension groups to explore the potential interaction effect.

All statistical analysis with data management was conducted by SAS version 9.4 (SAS Institute, Cary, North Carolina, USA). The significance was set at *P* < 0 0.05 with the two-tail test.

## Results

### Study population

**Fig. 1:**
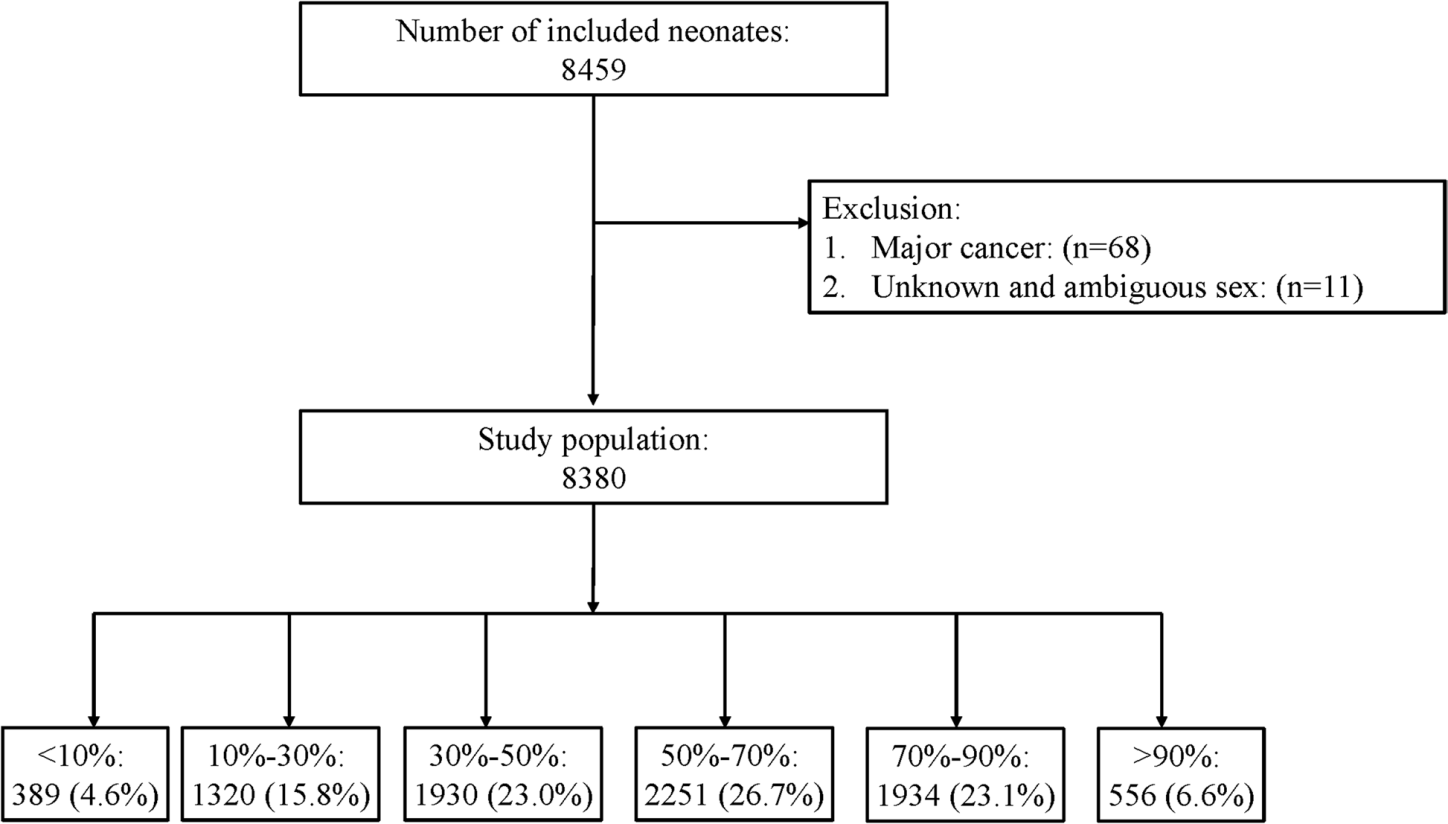
Flow chart of the study participants

A total of 8,380 participants were included in the study after excluding 79 participants who did not meet the criteria (Fig. 1). The median of gestational age and birth weight were 30 (28, 31) weeks and 1329.81 ± 319.50 g, respectively.

### Baseline characteristics

Table 1 presented the baseline characteristics of mothers and infants across percentiles of birth weight for gestational age. As the percentile of birth weight decreases, mothers tended to use steroid drugs (*P*_for trend_ <0.01) and tend to have more likely a cesarean section (*P*_for trend_ <0.01), to be primiparous (*P*_for trend_ <0.01), and to have hypertension (*P*_for trend_ <0.01)

**Table 1:** Maternal and infant’s characteristics for each birth weight-for-gestational age

Characteristics	Percentile of Birth Weight						Overall	*P*-Value
	< 10th	10th-30th	30th-50th	50th-70th	70th-90th	> 90th		
**N**	389	1320	1930	2251	1934	556	8380	
**Maternal Information**								
Maternal Age, Mean(std)	31.38 (4.84)	31.12 (4.95)	30.77 (5.07)	30.96 (4.85)	31.18 (4.84)	31.11 (5.28)	31.02 (4.94)	0.07
Antenatal Steriod Usage, N(%)	302/371 (81.4%)	1011/1255 (80.6%)	1417/1802 (78.6%)	1666/2098 (79.4%)	1344/1784 (75.3%)	357/509 (70.1%)	6097/7819 (78.0%)	< 0.01
Hypertension, N(%)	259/387 (66.9%)	626/1309 (47.8%)	373/1913 (19.5%)	179/2220 (8.1%)	111/1899 (5.8%)	38/545 (7.0%)	1586/8273 (19.2%)	< 0.01
Diabetes, N(%)	66/385 (17.1%)	204/1304 (15.6%)	306/1908 (16.0%)	395/2220 (17.8%)	346/1898 (18.2%)	130/548 (23.7%)	1447/8263 (17.5%)	< 0.01
Prenatal Visit > 1,N(%)	379/381 (99.5%)	1277/1285 (99.4%)	1852/1878 (98.6%)	2181/2202 (99.0%)	1855/1880 (98.7%)	528/536 (98.5%)	8072/8162 (98.9%)	0.19
C-section, N(%)	345/388 (88.9%)	1013/1318 (76.9%)	1081/1925 (56.2%)	1119/2244 (49.9%)	885/1922 (46.0%)	227/552 (41.1%)	4670/8349 (55.9%)	< 0.01
Primi-Gravida, N(%)	208/388 (53.6%)	718/1309 (54.9%)	1000/1921 (52.1%)	1127/2233 (50.5%)	938/1922 (48.8%)	261/552 (47.3%)	4252/8325 (51.1%)	< 0.01
Multiple Birth, N(%)	105/389 (27.0%)	373/1320 (28.3%)	642/1930 (33.3%)	713/2251 (31.7%)	581/1934 (30.0%)	114/556 (20.5%)	2528/8380 (30.2%)	< 0.01
**Infants Information**								
Gestational Age, Median(P25,P75)	30.86 (29.71,31.43)	30.43 (29.14,31.29)	30.00 (28.71,31.00)	29.86 (28.43,30.86)	29.43 (28.00,30.57)	29.29 (28.00,30.57)	30.00 (28.43,31.00)	< 0.01
<26weeks	8/389 (2.1%)	20/1320 (1.5%)	54/1930 (2.8%)	77/2251 (3.4%)	98/1934 (5.1%)	29/556 (5.2%)	286/8380 (3.4%)	< 0.01
26–27 weeks	26/389 (6.7%)	110/1320 (8.3%)	214/1930 (11.1%)	308/2251 (13.7%)	337/1934 (17.4%)	92/556 (16.5%)	1087/8380 (13.0%)	
28–29 weeks	76/389 (19.5%)	369/1320 (28.0%)	636/1930 (33.0%)	776/2251 (34.5%)	719/1934 (37.2%)	216/556 (38.8%)	2792/8380 (33.3%)	
30–31 weeks	279/389 (71.7%)	821/1320 (62.2%)	1026/1930 (53.2%)	1090/2251 (48.4%)	780/1934 (40.3%)	219/556 (39.4%)	4215/8380 (50.3%)	
Birth Weight, Mean(std)	917.98 (182.91)	1128.78 (211.02)	1262.44 (244.97)	1371.69 (273.64)	1466.98 (309.60)	1682.40 (371.42)	1329.81 (319.50)	< 0.01
Male, N(%)	237/389 (60.9%)	755/1320 (57.2%)	1017/1930 (52.7%)	1286/2251 (57.1%)	1060/1934 (54.8%)	344/556 (61.9%)	4699/8380 (56.1%)	< 0.01
Apgar score < 7 at 5 min, N(%)	34/370 (9.2%)	76/1270 (6.0%)	119/1841 (6.5%)	135/2150 (6.3%)	134/1825 (7.3%)	40/517 (7.7%)	538/7973 (6.7%)	0.18
Inborn, N(%)	295/389 (75.8%)	974/1320 (73.8%)	1362/1930 (70.6%)	1619/2251 (71.9%)	1330/1934 (68.8%)	360/556 (64.7%)	5940/8380 (70.9%)	< 0.01

### The influence of birth weight for gestational age on neonatal outcomes

**Fig. 2:**
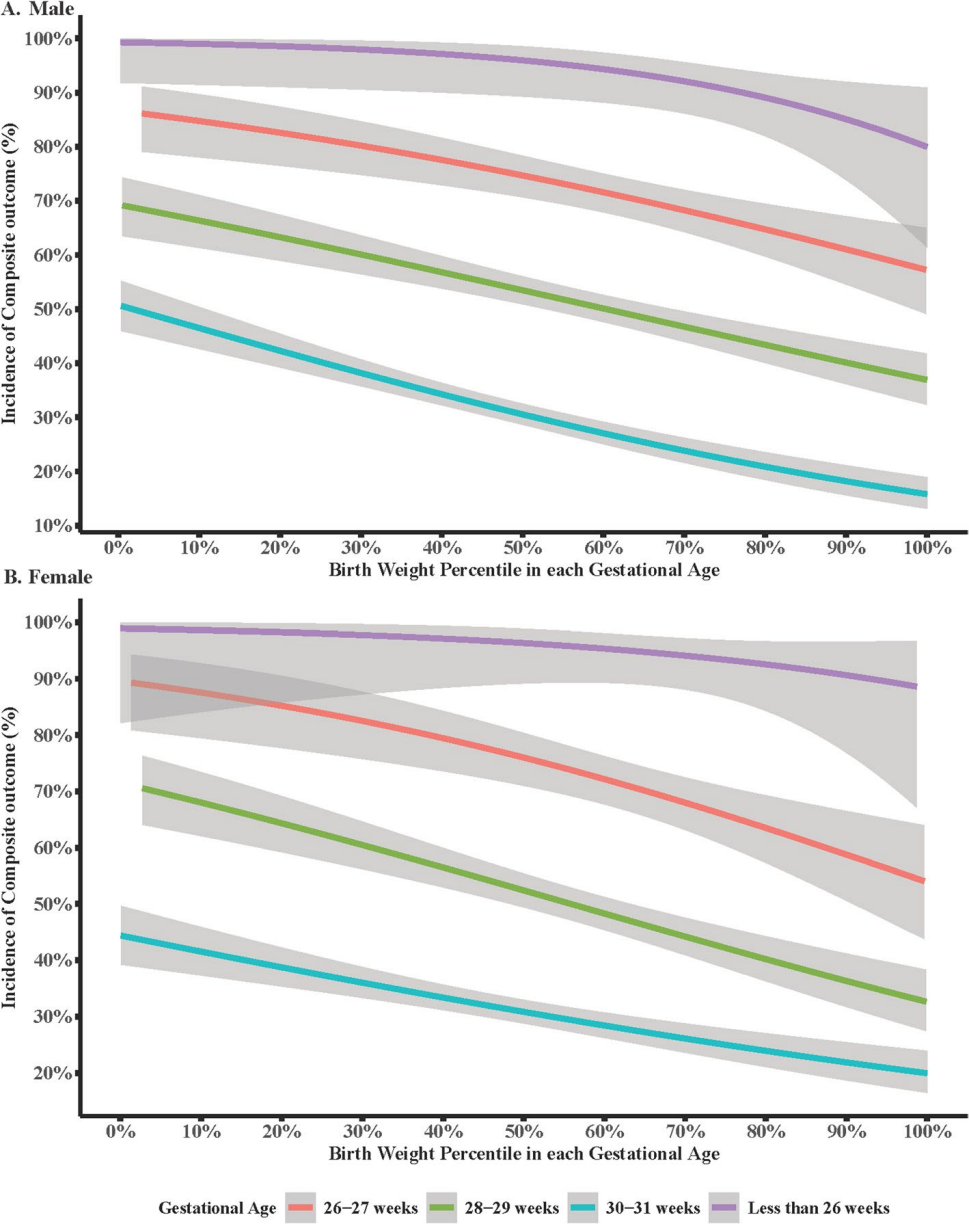
Regression analysis of the association between birth weight for gestational age and adverse neonatal outcome

Table 2 presented the incidence of neonatal outcomes in each birth weight-for gestational age group. The total incidence of composite outcomes was 45.1% within the study population. As the birth weight percentile increased, the incidence of composite outcome decreased gradually with *P*_for trend_<0.01. This trend was also observed in regression analysis, especially at gestational age of 26–27 weeks, 28–29 weeks, and 30–31 weeks (Fig. 2). Moreover, the incidence of mortality (*P*_for trend_ <0.01), severe BPD (*P*_for trend_ <0.01), sepsis (*P*_for trend_=0.02), and severe ROP (*P*_for trend_=0.03) also decreased when a percentile of birth weight increased.

**Table 2:** Incidence of neonatal outcome in each birth weight-for-gestational age

Outcome, *n*/*N*(%)	Percentile of Birth Weight						Overall	*P*-Value for Trend
	< 10th	10tht-30 h	30th-50th	50th-70th	70th-90th	> 90th		
**Number**	389	1320	1930	2251	1934	556	8380	
**Composite Outcome**	249/389 (64.0%)	679/1320 (51.4%)	886/1930 (45.9%)	924/2251 (41.0%)	819/1934 (42.3%)	219/556 (39.4%)	3776/8380 (45.1%)	< 0.01
Mortality	79/389 (20.3%)	170/1320 (12.9%)	224/1930 (11.6%)	228/2251 (10.1%)	245/1934 (12.7%)	61/556 (11.0%)	1007/8380 (12.0%)	< 0.01
NEC ≥ Stage II	32/389 (8.2%)	66/1320 (5.0%)	75/1930 (3.9%)	107/2251 (4.8%)	88/1934 (4.6%)	24/556 (4.3%)	392/8380 (4.7%)	0.1
Severe BPD	198/389 (50.9%)	524/1320 (39.7%)	652/1930 (33.8%)	644/2251 (28.6%)	595/1934 (30.8%)	143/556 (25.7%)	2756/8380 (32.9%)	< 0.01
Brain Damage ^a^	32/323 (9.9%)	111/1100 (10.1%)	165/1639 (10.1%)	211/1947 (10.8%)	192/1657 (11.6%)	58/477 (12.2%)	769/7143 (10.8%)	0.07
Severe IVH	23/324 (7.1%)	75/1093 (6.9%)	101/1631 (6.2%)	129/1934 (6.7%)	138/1649 (8.4%)	42/473 (8.9%)	508/7104 (7.2%)	0.04
cPVL	16/331 (4.8%)	53/1144 (4.6%)	83/1697 (4.9%)	108/2002 (5.4%)	91/1719 (5.3%)	28/489 (5.7%)	379/7382 (5.1%)	0.27
Severe ROP ^b^	18/288 (6.3%)	42/1055 (4.0%)	47/1520 (3.1%)	52/1733 (3.0%)	49/1439 (3.4%)	9/387 (2.3%)	217/6422 (3.4%)	0.03
Sepsis	40/360 (11.1%)	123/1251 (9.8%)	180/1848 (9.7%)	186/2155 (8.6%)	164/1843 (8.9%)	38/532 (7.1%)	731/7989 (9.2%)	0.02

*Abbreviations*: *NEC* Necrotizing enterocolitis, *BPD* Bronchopulmonary dysplasia, *IVH* Intraventricular hemorrhage, *cPVL* cystic periventricular leukomalacia, *ROP* Retinopathy of prematurity

^a^Incidence of brain injury was calculated among infants with neuroimaging results

^b^Incidence of severe ROP was calculated among infants with eye examinations in the NICU

**Fig. 3:**
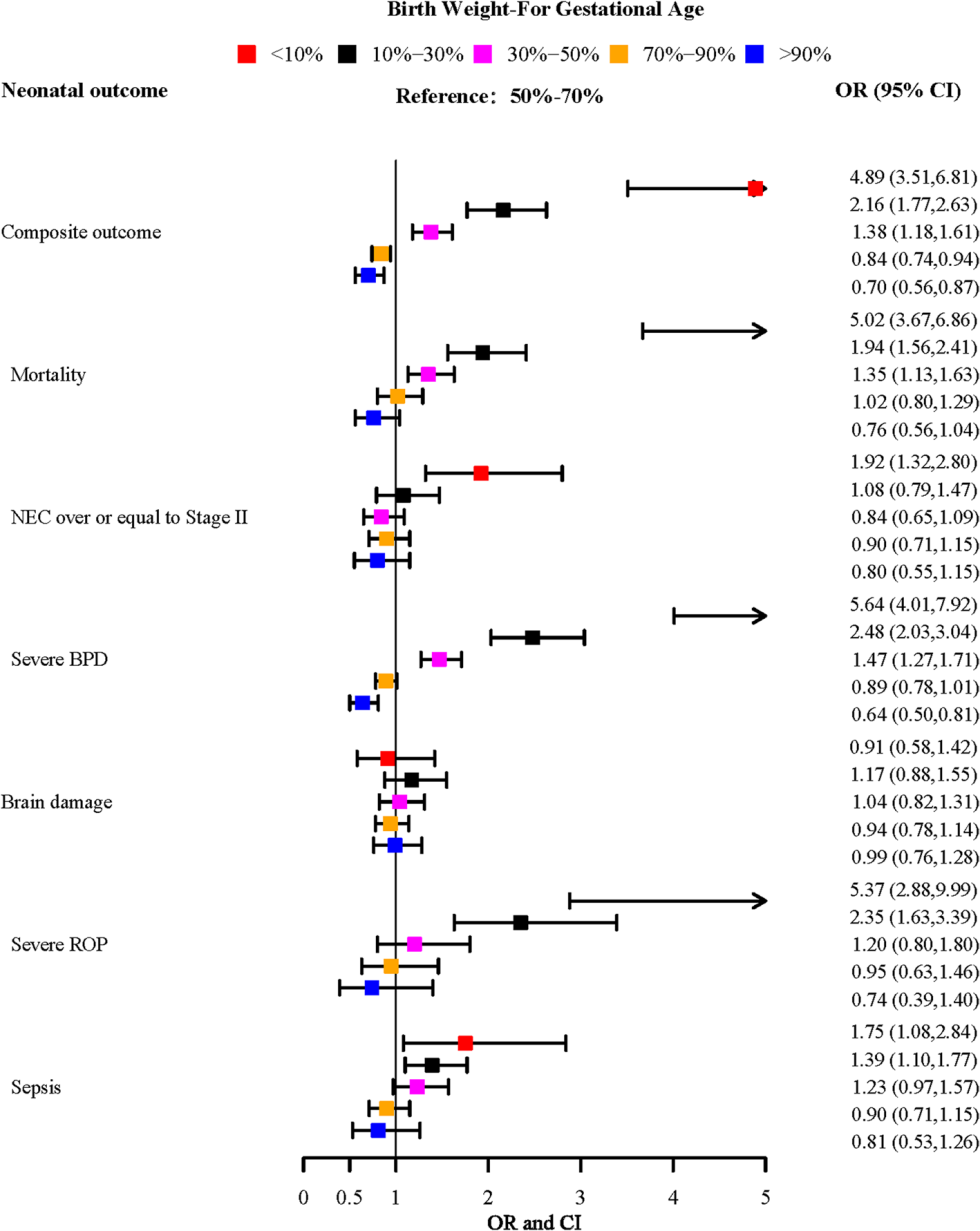
Forest plot of the association between birth weight for gestational age and adverse neonatal outcome in total participants

Our results indicated that the risk of composite outcome was negatively related to the percentile of birth weight-for gestational age. As shown in Fig. 3, compared to the reference group (50th -70th percentile of birth weight for gestational age), the ORs (95%CI) of composite outcome, mortality, severe BPD, severe ROP, and sepsis for 10th -30th percentile of birth weight for gestational age was 2.16 (1.77–2.63), 1.94 (1.56–2.41), 2.48 (2.03–3.04), 2.35 (1.63–3.39), and 1.39 (1.10–1.77), respectively. In the stratified analysis by gestational age, our results remain stable in the 28–31 weeks group (Supplemental Fig. 1), except for sepsis. In the stratification analysis by maternal hypertension, there was a significant association between birth weight for gestational age < 30th percentile and risk of mortality, BPD, and ROP among non-maternal hypertension infants. However, the risk of mortality, ROP, and NEC was only significant in maternal hypertension infants with birth weight < 10th percentile (Supplementary Fig. 2). In addition, compared to the reference group, we also observed an increased risk of composite outcome, mortality, and severe BPD in newborns with birth weight at 30th -50th percentiles. The corresponding ORs (95% CIs) were 1.38 (1.18–1.61), 1.35 (1.13–1.63), and 1.47 (1.27–1.71), respectively.

## Discussion

To the best of our knowledge, this is one of the largest studies to explore the relationship between the percentile of birth weight for gestational age and adverse neonatal outcomes among Chinese NICUs. In this study, SGA was related to composite outcomes and mortality. Furthermore, infants with a birthweight < 25th percentile had a higher risk of death and BPD, ROP, and sepsis than infants at the 50-75th percentile.

The adverse outcomes of newborns with abnormal fetal growth, especially morbidity and mortality, are of great concern to medical staff, parents, and health care systems. Several studies have examined the morbidity and mortality of SGA infants over time. Despite controversy, most studies suggested that SGA neonates appear to be at greater risk for intrauterine fetal death (IUFD), perinatal death, and other adverse outcomes than non-SGA neonates [27–29]. More importantly, several prior studies have consistently indicated that, in the case of preterm newborns, the threshold for increased risk of morbidity and mortality does not align with the 10th percentile but tends to be at a higher percentile [8, 9, 11]. Despite the utilization of different charts for birth weight percentiles across studies conducted in different countries, the results and conclusions have demonstrated high consistency. Our study’s findings, utilizing a study population of Chinese very preterm infants, further align with those of previous studies.

In this present study, we found that preterm infants considered appropriate for gestational age, such as infants with a birthweight within 10th -30th percentile, were at increased risk of morbidities and mortality. There was a continuous decline trend in incidence of composite outcomes with the increasing birthweight for gestational age. Potential mechanisms may be placenta lesions or immaturity of metabolic pathways. A previous study found that compared with the AGA newborn, most metabolites increased in SGA group and decreased in LGA group. Such changes may be influenced by placental factors and may be related to long-term outcomes of neonates [30]. The detailed mechanism for low birthweight and BPD is not clearly established. However, several mechanisms could be proposed. Lal et al. found that SGA preterm infants are at higher risk of BPD and LGA infants have a reduced incidence of BPD [31]. Experiments suggested that fetal growth restriction led to organic lesion in the lung, including a thicker pulmonary blood-air barrier, smaller number of alveoli, and thicker interalveolar septa, which might impair gas exchange and alter the mechanical properties of the lungs [32]. Importantly, our study revealed that newborns with birth weights in the 30th -50th percentiles also exhibit elevated risks of mortality and severe BPD. This suggests that the impact of low birth weight on neonatal mortality and the risk of severe BPD is even more substantial.

Studies believe that low birthweight is risk factor for ROP [33]. Garite et al. showed IUGR was associated with increased incidence of ROP [29]. This is consistent with our conclusion. In contrast, a previous study has shown a low incidence of ROP (0.5%) at a birth weight of less than 1500 g [34]. A large population-based study in Sweden found that preterm delivery was the most important risk factor for ROP, and there was no association between SGA and risk of ROP [35]. Potential reasons for this discrepancy may be due to differences in sample size, as well as in infants’ characteristics.

As expected, we observed a significant relation between birth weight and maternal hypertension. In this present study, mothers were more likely to have hypertension with the birth weight% less than 30th, consistent with previous findings. We performed stratification analysis by maternal hypertension, which made it possible to explore our hypothesis whether there was an interaction effect between etiologies of very preterm birth, such as maternal hypertension, and the impact of growth restriction. There were discrepant results, that we above mentioned, among maternal and no maternal hypertension groups. For mortality risk, one possible reason for the discrepancy is the difference in mortality risk between infants with or without maternal hypertension. Previous studies indicated that preterm infants from non-maternal hypertension pregnancies have a higher mortality risk than infants from pregnancies complicated by maternal hypertension [36–38]. In our study, we also observed this phenomenon. The mortality risk was higher in the non-maternal hypertension group than the maternal hypertension group, especially in infants with birthweight less than 30th percentile (15.7% versus 13.7%). Although the detailed mechanisms remain unclear, they likely involve placental perfusion. Although most of the maternal hypertension cases may not fit the hypoperfusion model, it might improve the overall placental perfusion, which could lead to the appropriate and increased fetal growth [36, 39]. Future research is required to confirm our conclusion and investigate the mechanisms underlying this discrepancy.

### Strengths and limitations

There were several strengths of our study. A large multi-center cohort design assures enough sample size to provide estimates of association for our primary outcome. Detailed birth weights for gestational categories ensure more information is provided. There are several limitations. Firstly, the weighting scales from participating centers were hard to thoroughly correct, which may lead to bias from the actual weight. Secondly, the data are from a select group of large tertiary NICUs with the highest level of neonatal care in China and may not be representative of the general population. Thirdly, reflecting fetal growth restriction solely through birth weight percentiles may have limitations, as it does not account for other obstetric data, such as anthropometric measurements or Doppler measurements. Future research should consider obstetric data for a more comprehensive assessment. Fourthly, our study did not include delivery room deaths and infants not admitted to NICUs, albeit a small proportion of these conditions has been observed. Fifthly, since not all cases of neonatal sepsis exhibit positive blood or cerebrospinal fluid cultures, future research should consider including inflammation markers. Finally, our research primarily focuses on low birth weight for gestational age rather than congenital defects or alterations, which can also lead to growth restriction [40–43]. Future research might focus on low-birth weight or fetal growth impairment resulting from congenital defects or alterations, with the aim of providing the better possibility of care to all children.

## Conclusion

Birth weight for gestational age below the 30th percentile may have a significant negative impact on neonatal outcomes. Regular monitoring and early intervention are crucial for these high-risk infants. By enhancing follow-up care, we can promptly identify and manage potential health issues, thereby improving the long-term outcomes of these infants. Additionally, prevention strategies should not only apply to infants with low birth weight due to prematurity but also extend to other categories of infants with low birth weight or fetal growth impairment resulting from congenital defects.

## Supplementary Information

Supplementary Material 1.

Supplementary Material 2.

## Data Availability

Due to the nature of this research, participants of this study did not agree for their data to be shared publicly, so supporting data is not available.
