# Supplementary figures and images for: Association of neonatal outcome with birth weight for gestational age in Chinese very preterm infants: a retrospective cohort study
Source: Ital J Pediatr. 2024 Oct 4;50:203. doi: 10.1186/s13052-024-01747-1 (PMC11451004; doi:10.1186/s13052-024-01747-1)

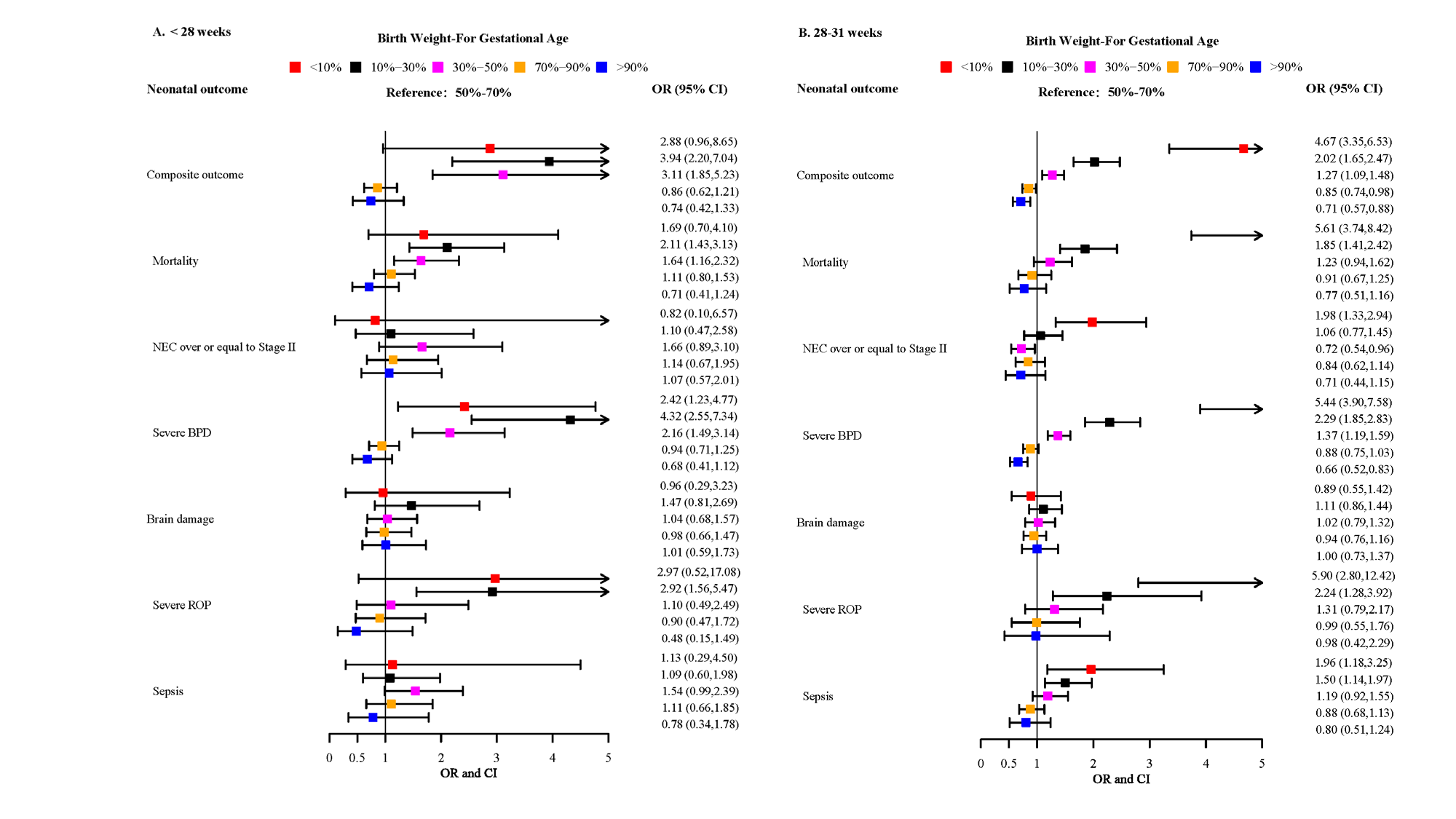

Supplement: Supplementary file 1 — Supplementary Material 1. [file 13052_2024_1747_MOESM1_ESM.png]

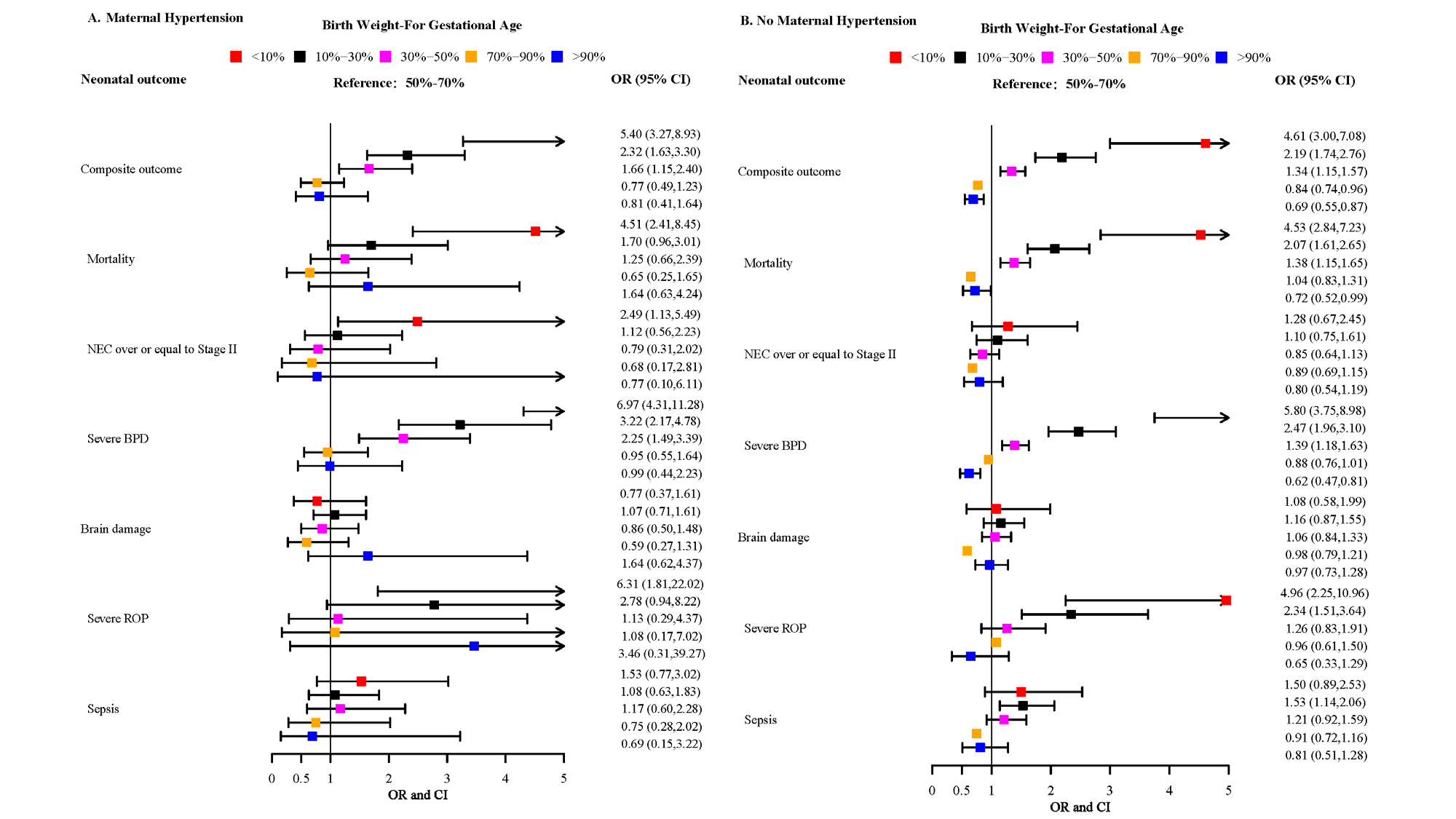

Supplement: Supplementary file 2 — Supplementary Material 2. [file 13052_2024_1747_MOESM2_ESM.png]
